# Address to the Graduating Class of the Medical Department of Niagara University

**Published:** 1886-05

**Authors:** Simeon T. Clark

**Affiliations:** Prof. of Medical Jurisprudence


THE BUFFALO MEDICAL AND SURGICAL JOURNAL

Vol. XXVI. MAY, 1886. No. 10.

### Original Communications.

### *Address to the Graduating Class of the Medical Department of Niagara University.

By Simeon T. Clark, A. M., M. D., Prof. of Medical Jurisprudence.

*Most Honored Chancellor, Respected Colleagues of the Medical Department of Niagara University—Ladies and Gentlemen:*

I am not unmindful of the great honor conferred on me at this time; but I assure you all it was as unsought by me as unexpected. It devolves on me, not by merit or fitness, but in a necessary division of labor. My more accomplished brethren, who have wrought so earnestly and faithfully to render this occasion—what all will allow it has been thus far—a dignified and impressive scene, could not bear all the burdens; neither did it seem fitting that one who had not been engaged in teaching this class should be called to counsel "Our Boys" on this, the most eventful evening of their lives. Therefore, O Venerable Prelate! may we not be permitted for "these few fleeting moments," "these brief sands of time," to forget that our homely sentences are to be spoken in your presence and hearing? If duty imperative did not kindly furnish its anæsthetic to

*Delivered at the Annual Commencement.

benumb, in part, our sensibilities, we should be dumb in the presence of one whose lips have been touched as by a live coal from off the altar of inspiration, and whose words have been fittingly compared to "apples of gold in pictures of silver."

I am sure no one of my co-workers will attempt to disprove the power vested in the serpent-twined staff of Æsculapius, to render invisible, for half an hour, even these mighty medical giants, if only in imagination this ancient caduceus be waved in the manner of the conjurors and soothsayers of old—above their gowns and hoods. But it is not sufficient to render ourselves oblivious to Chancellor and Faculty. Here is a goodly company of as true and trusty friends as ever rang a bell at midnight to summon a physician to duty. These are they to whom we owe our professional reputations and the pecuniary means of life. It may be that their first impression of a doctor is embittered by the memory of quinine, and unctious with a lingering recollection of castor-oil. In their nomenclature, the medical profession is divided into two classes, both of an ornithological nature. The irregulars are placed in the order *natatores*, family *anatidæ*, a class that proclaims its character in its one unmusical cry, quack, quack! The regulars are elevated into the order—*grallæ*, of which the woodcock and plover are representatives. And these are particularly distinguished on account of their unusually wise look and still more uncommonly long bills.

But these patrons are not alone. Beside them are influences more potent than chancellors, faculties, or courtly friends—the mothers, wives, sisters and daughters of our hearts' best love—a passion little less than worship. These have left the duties and delights of home to bring to this scene the charm of their presence. Sharply defined in our memories is the place assigned to woman by the Egyptians, when they were the wisest of the nations of the earth. The spirit of wisdom was given the form of a beautiful matron, who held in her shapely hand a weaver's shuttle. This form, in time, became the cloud-draped, olive-crowned Athenea of the Greeks, to whom we owe so much that is æsthetical in literature and art. Woman, the crowning work of creation! The blue of the violet pales before the azure of her eyes; the rose's red is but a faint reflection of her blushes; and the lily's pure whiteness lacks the soft velvety warmth of her spotless brow. To this involution of physical charms are added the benison of all womanly virtues, and the adornments of mind and refinements of soul that enable her to supplement the arc of man's existence, and complete the circle of human perfection. She weeps over our fallen natures, and the seeds of virtue spring up in our barren souls; and in the sunshine of her smiles these grow into fadeless amaranths to gladden the eternal gardens of the King of Glory!

On this occasion we must not yield to the power of wisdom, strength or beauty. Forgetting Chancellor, Faculty, Knights and Ladies, we desire to stand alone with the Graduating Class of the Medical Department of Niagara University.

One hour ago, gentlemen, you were medical students; now, I address you by the distinguished title of Doctors of Medicine; but I give it you strictly in charge that you still remain and ever continue students of medicine.

Lord Bacon says: "The greatest trust between man and man is the trust of giving and receiving counsel. The wisest princes need not think it any diminution of their greatness, or derogatory to their sufficiency, to rely upon counsel. God Himself is not without it, but hath made one of the great names of the blessed and co-equal Son—the Counsellor."

When in the sanctity and secrecy of their several lecture-rooms your professors, each in his appropriate department, have given you of the fullness of their knowledge and experience, they have been your counsellors, and this evening they have deputised me to sum up the whole matter—how you shall best add strength to the noble profession you have chosen, honor to your Alma Mater, who will always, in the future, as she has in the past, prove a kind mother to all, and, finally, adorn your lives with the grandest of human achievements—an honorable and distinguished name. One thing will always remain to you that no other class can ever attain to. You are, and will be henceforth and forever more, the first class ever graduated from the Medical Department of Niagara University. This class, together with the professors and lecturers who have received the gown and hood, constitute the nucleus of the Alumni Association of this College. When we consider the high plane on which this University has been placed, the extended and liberal course of instruction which will be demanded, yea, *exacted*, of all who succeed you, there is fit reason to be proud to stand as the illustrious predecessors of such a noble army as will surely follow you. There is something of intrinsic value associated with the first of all acts or endeavors. Think how the poets have hallowed this sentiment:—

> "Fresh as the *first* beam glittering on a sail."
>
> "Deep as *first* love, and wild with all regret."
>
> "Fierce as the *first* shock of contending foes."

You are the *first* fruits—the *first* born of the Medical Department of Niagara University. As such I congratulate you, not only that you have been found worthy to receive this degree, but that you have attained it after passing a test examination as severe, I believe, as was ever given a graduating class on this side of the Atlantic.

To the average medical student, on the eve of his graduation, a mythological allusion would be lost, but it will be patent to you, who, long before you knocked at the outer door of the temple of medicine, had wandered, by the light of the midnight oil, through the intricate and winding labyrinths of integral and differential calculus, and had felt your souls grow harmonious at the bidding of Homer and Virgil. You well remember that Apollo, the son of mighty Jove, King of all the Grecian deities, was the god, not only of music, painting and sculpture, but also of medicine. The son of Apollo, by Koronis, daughter of a Thessalian Prince, was named Æsculapius, and he was, and is, styled the demi-god of medicine, as Hippocrates is the father. To Æsculapius the number seven was sacred. His temples of worship, which afterwards became hospitals, were built in the form of heptagons, or seven-sided figures. On the seven sided altars, seven unplucked fowls were sacrificed to dissipate pestilence, and propitiate the favor of this god. May we not congratulate you and the Medical Department of Niagara University, that this, the first class asking for graduation, was of this mystical number seven; not only sacred to Æsculapius, but suggestive of the seven liberal arts and sciences, the seven wonders of the world, and that beautiful constellation the Pleiades, or seven stars. For though, alas! one of your number was plucked, he may now typify "the lost pleiad," and you may still make these bright orbs of heaven the jewels of your class. Bind their sweet influence to your imperial ægis. They are, as you were, seven, and when—

> "In the night you see the Pleiades rising through the mellow shade,
> Glittering like a swarm of fire-flies tangled in a silver braid,"

remember that as our world and her sister worlds are revolving about the sun, that same sun, and the system of suns to which he belongs, are revolving about your constellation—that Alcyone, the brightest of the seven, is the central sun to which our own day-king bows! May we not hope to carry this comparison still further? Why may not the most brilliant of this group of medical stars become so eminent in his profession that the medical world shall make him their central light and controlling power?

And now, gentlemen, I desire to speak to you of the significance of the rites and ceremonies through which you have just passed. In adopting the form of graduation peculiar to Oxford and Cambridge, we may be accused of aping effete monarchies; others may go so far as to call these forms an outgrowth of Romanism. Nothing could be more foreign to the fact, or could more fully display the ignorance of such as may condescend to make this or kindred criticisms.

These are the historical and classical illusions to which our rites point, and from which they derive significance:

In the middle ages, Professors of, and Doctors in Divinity, Medicine, Laws and Arts were directed to wear long, loose, flowing robes, to distinguish them from men-at arms, knights and their squires, who were not only fettered by ignorance, but still farther hampered by unwieldy coats-of-mail. As learning was revived and became more common, all the students in the several departments of the universities were permitted to wear the gown, so emblematical of their freedom from ignorance and the quixotic follies of knight-errantry. At this time it became necessary to designate such as had passed through the required grades of study from those who were yet in their pupilage. Therefore, after the seventeenth century, Oxford decreed that a hood should be added to the scholarly costume, as a badge of distinction of the several learned professions and their different grades. The hood of a Bachelor of Arts, Law or Medicine had but one cauda, to show he had yet one step to advance. The divinity-hood was of black, lined with white, with one or two caudæ, as he was Bachelor or Doctor of Divinity. The Bachelor of Arts wore a black hood, with a narrow edge of ermine. The Master of Arts had a lining of crimson or cherry-colored satin. The Doctor of Laws wore a hood of white satin, lined with purple, the white to show the purity of the law, and the purple lining, being the blending of red, the color of blood, and blue, the color of mercy, was to show that mercy should ever temper the sword of justice. The hood of the Doctor of Medicine you have seen, yea, have received. It is of red, as you behold — red, the emblem of life, as it is so closely allied to the color of the sun and of fire, which, to the Ancients, were the great life-generating, life-preserving powers in the earth. The lining, you observe, is blue, the color of truth and of mercy; it should teach the wearer that mercy and truth should govern the doctor, not only in shedding the blood of his patients, but in his defense of the faith.

But still more is signified by the Doctor's hood. The first statue, found in the sculptures of the Ancients, wearing a hood, was that of Telesphorus, the son of Æsculapius, the genius or deity that presided over that secret and mysterious vitality which sustains the convalescent. He was always represented on the left of Æsculapius, as Hygiea, the goddess of health, was on the right. He was closely wrapped in his gown, and hooded. The careful wrapping up seems to indicate the shrouded nature of the vital force which he personifies, and the care which should be given ever to all convalescents. His is the first hood on record, and is the model from which all others have been copied. Whenever your gown and hood are derided, invoke the spirit of Telesphorus, the deity of convalescence, for your warrant, and the scholars of all ages will acknowledge your kinship. Your university degree, of which the gown and hood are the emblems, will be to you what the golden branch plucked from the sibyline tree was to "the pious Æneas," who would have failed in his dreadful enterprise of invading the realm of the dead without it; for you, too, must go down with your patients almost to the valley and shadow of death; and, as by the same means he vanquished the three-headed dog, Cerberus, so must you overcome the many headed hydra of disease. This golden branch was held out to Charon, and he dare not refuse to ferry a living mortal across the Stygian tide. Your degree will be a passport into the convocations of the wisest and best men of all nations; for the learned have ever held that education is of little or no value, only so far as it conduces to a kingly power over yourselves, and through you to others of your kind, onward and forward to the last moment of human necessity, and, as it enables the possessor understandingly to consult with the great, the wise and the good of all ages. "Books will speak plain, when counsellors turn pale." The dead are the best counsellors of the living; they give, but never receive.

It is the fullness of the treasure of books, and the superiority of inventions, that makes you to night—

> "Heirs of all the ages, in the foremost files of time."

And now, gentlemen, a few mentions of what you must not do. There is a no little danger that your professional usefulness may be early impaired by the demon of pride taking you up into an exceeding high mountain, and showing you all the kingdoms of this world, and assuring you of the most masterly success if you will consent to make this your dwelling-place; in other words, become a specialist from the beginning of your professional career. I have no doubt that after a dozen years or so of general practice many of you will naturally gravitate toward some special branch of practice; that is legitimate, but, until after attaining an all-sided professional character in general practice, you are forced, by environing circumstances and the natural adaptation of your talents, and the almost unanimous verdict of your professional brethren, to take such a course, avoid it as you would the dismemberment of your bodies, or the loss of some attribute of the mind.

Again, never forget that the status of a professional man is not his own estimate of his value. No better speculation could be entered into than buying certain professional men at their true value, and selling them at their own valuation of themselves—the profit would be enormous. It is just as true that the position to which the public assigns them is often faulty. Communities often over and underrate us. The true place which you or I, as doctors, must find at last is the nitch to which the profession assigns us. The valuation which our professional brethren place upon us is correct, and the sooner we admit the fact the better for our happiness.

Again, gentlemen, remember, "Honor to whom honor is due." When you locate in some new field of practice do not expect to rise by the mistakes of others, or by climbing a ladder, every round of which is the blasted reputation of some other doctor. If you do, you will fall to the ground, no matter to what height you attain, and you will deserve the fall. If you cannot speak well of your fellows in the profession, keep silent. Let your work and not your words advertise you. Finally, keep this principle in mind always in the treatment of disease: Follow the leading of nature; this is the pole-star of successful practice. Do not claim to cure disease, but instruct the families into which you are called, as speedily as possible, concerning the relation which a true physician occupies in the management of disease. Teach them the self-limited nature of many morbid conditions, and how little curative power drugs possess. Show to them that as the pilot who is powerless to quell the storm, is still able to guide the ship to safety, so the skillful physician prevents the wreck of human life, as he carefully watches the progress of disease, guarding against the approach of death at this point, and at the other shutting a sluice-way from which life is in danger of escaping.

If you know anything about the case you are treating, be not afraid to instruct and make plain to all interested the nature of the departure from the physiological condition, and the course you intend to pursue to bring this pathological state back to the condition of health.

"Be instant in season and out of season" to impress this truth on the minds of men—that it is impossible for the doctor to give health to those who are daily transgressing the laws of being, and still more impossible to make strong and perfect bodies for such as are reeking in the consequences of the sins of generations before them.

This leads us to speak of the preventive medication, or the preventive practice of the future. It is my desire that every graduate from this institution shall constitute himself a committee of one to labor most heartily to bring in the glorious day when it shall be the duty of the physician to keep the people in health, rather than restore to health the sick. Not only must you study with your microscope the secretions of the body, but the air your patients breathe, the food they eat, the water they drink, and even the paper on the walls where they live. The doctor of to-day should know as much about the relations of sewer-traps as of stethoscopes, and labor more earnestly to prevent epidemics than to suppress them.

Finally, gentlemen, you must labor in the practice of your profession as you have in the attaining of it. Remember the true physician is first learned, then brave, next generous, chaste and pious—without these *ad eundem* qualifications, your degree will be of little worth; but, if you add to your knowledge virtue, and unite temperance, chastity and faith, then, when it is said, as it will one day be said of each of you, "Doctor is dead," may the same sweet influence gather about your names as to-day adorns the memory of the great and good man who welcomed you in the beginning of your professional career, but who, before you received the honors of this evening, had himself taken, at the hand of the Almighty and All-glorious Chancellor of the Universe, the last and highest degree attainable—that of a just man made perfect in the Kingdom of our God. The crowning glory of Professor Charles C. F. Gay was not his professional skill, which was acknowledged; but his most kind and loving bearing towards his professional brethren. When at last the purple current curdles in your veins; when your hearts cease to beat, and you gather up your feet to die,—may those who knew you best mourn you most deeply, and this be said of each of you: "He was a wise, skillful and conscientious physician."

